# The Severe Acute Respiratory Syndrome (SARS)-coronavirus 3a protein may function as a modulator of the trafficking properties of the spike protein

**DOI:** 10.1186/1743-422X-2-5

**Published:** 2005-02-10

**Authors:** Yee-Joo Tan

**Affiliations:** 1 Institute of Molecular and Cell Biology, 61 Biopolis Drive, Proteos, 138673 Singapore

## Abstract

**Background:**

A recent publication reported that a tyrosine-dependent sorting signal, present in cytoplasmic tail of the spike protein of most coronaviruses, mediates the intracellular retention of the spike protein. This motif is missing from the spike protein of the severe acute respiratory syndrome-coronavirus (SARS-CoV), resulting in high level of surface expression of the spike protein when it is expressed on its own *in vitro*.

**Presentation of the hypothesis:**

It has been shown that the severe acute respiratory syndrome-coronavirus genome contains open reading frames that encode for proteins with no homologue in other coronaviruses. One of them is the 3a protein, which is expressed during infection *in vitro *and *in vivo*. The 3a protein, which contains a tyrosine-dependent sorting signal in its cytoplasmic domain, is expressed on the cell surface and can undergo internalization. In addition, 3a can bind to the spike protein and through this interaction, it may be able to cause the spike protein to become internalized, resulting in a decrease in its surface expression.

**Testing the hypothesis:**

The effects of 3a on the internalization of cell surface spike protein can be examined biochemically and the significance of the interplay between these two viral proteins during viral infection can be studied using reverse genetics methodology.

**Implication of the hypothesis:**

If this hypothesis is proven, it will indicate that the severe acute respiratory syndrome-coronavirus modulates the surface expression of the spike protein via a different mechanism from other coronaviruses. The interaction between 3a and S, which are expressed from separate subgenomic RNA, would be important for controlling the trafficking properties of S. The cell surface expression of S in infected cells significantly impacts viral assembly, viral spread and viral pathogenesis. Modulation by this unique pathway could confer certain advantages during the replication of the severe acute respiratory syndrome-coronavirus.

## Background

The recent severe acute respiratory syndrome (SARS) epidemic, which affected over 30 countries, resulted in more than 8000 cases of infection and more than 800 fatalities (World Health Organization, ). A novel coronavirus was identified as the aetiological agent of SARS [1]. Analysis of the nucleotide sequence of this novel SARS coronavirus (SARS-CoV) showed that the viral genome is nearly 30 kb in length and contains 14 potential open reading frames (ORFs) [2-4]. These viral proteins can be broadly classified into 3 groups; (i) the replicase 1a/1b gene products which are important for viral replication, (ii) the structural proteins, spike (S), nucleocapsid (N), membrane (M) and envelope (E), which have homologues in all known coronaviruses, and are important for viral assembly, and (iii) the "accessory" proteins that are specifically encoded by SARS-CoV. Much progress have been made in characterizing these SARS-CoV proteins [5,6], but the molecular determinant for the severe clinical manifestations of SARS-CoV infection in contrast to the mild diseases caused by most coronaviruses, remains to be determined. In addition, the exact roles of "accessory" proteins of SARS-CoV are still poorly understood.

The subject of this hypothesis relate to the S protein and one of the "accessory" proteins, the SARS-CoV 3a protein. The S protein, which forms morphologically characteristic projections on the virion surface, mediates binding to cellular receptor and the fusion of viral and host membranes, both of these processes being critical for virus entry into host cells [7,8]. As such, S is known to be responsible for inducing host immune responses and virus neutralization by antibodies [9,10]. 3a (also termed ORF3 in [2] and [11], as X1 in [3], and as U274 in [12,13]) is the largest "accessory" protein of SARS-CoV, consisting of 274 amino acids and 3 putative transmembrane domains. Three groups independently reported the expression of 3a in SARS-CoV infected cells [13-15] and it was also detected in a SARS-CoV infected patient's lung specimen [14]. Antibodies against 3a were also found in convalescent patients [11,12,14].

This article hypotheses that the endocytotic properties of 3a allow it to modulate the surface expression of S and explores a functional significance for the interaction between S and 3a, which has been observed experimentally [13,15].

## Presentation of the hypothesis

The cellular fate of the S protein has been well mapped [16,17]: S is cotranslationally glycosylated and oligomerized at the endoplasmic reticulum. Its N-linked high mannose side chains are trimmed, modified and become endoglycosidase H-resistant during the transportation to the Golgi apparatus. Only this fully-matured form of S can be assembled into virions and/or transported to the cell surface. The latter could cause cell-cell fusion and the formation of syncytia. Recently, Schwegmann-Wessels and co-worker reported that a novel sorting signal for intracellular localization is present in the S protein of most coronaviruses, but absent from SARS-CoV S [18]. Site-directed mutagenesis studies confirmed that a YxxΦ motif (where x is any amino acid and Φ is an amino acid with a bulky hydrophobic side chain) retains the S protein of TGEV intracellularly when it is expressed alone. On the other hand, SARS-CoV S is transported efficiently to the cell surface unless such a motif is introduced into its cytoplasmic tail by mutagenesis.

The YxxΦ motif has been implicated in directing protein localization to various intracellular compartments [19-21]. Furthermore, most YxxΦ motifs are capable of mediating rapid internalization from the plasma membrane into the endosomes. Interaction between the adaptor protein complex 2 (AP-2) with the YxxΦ motif present in the cytoplasmic domain of the internalizing protein concentrated the protein in clathrin-coated vesicle, which then budded from the plasma membrane resulting in internalization. However, it appears that the YxxΦ motif can also bind other adaptor protein complexes, like AP-1, 3 and 4, and the differential binding to the different adaptors will determine the pathway of a cargo protein containing a particular YxxΦ motif [21]. Coincidently, a YxxΦ motif in the cytoplasmic domain of 3a has previously been identified [13]. Furthermore, the juxtaposition of the YxxΦ motif and a ExD (diacidic) motif was found to be essential for the transport of 3a to the cell surface, consistent with the role of these motifs in the transportation of other proteins to the plasma membrane [22]. 3a on the cell surface can also undergo internalization [13].

Analyzing the experimental results present in these publications collectively, it is possible to postulate a functional role for the evolution of the SARS-CoV 3a protein. The SARS-CoV S protein lacks the YxxΦ motif but it can bind to the 3a protein which has internalization properties. In SARS-CoV infected cells, S is rapidly transported to the cell surface. But if 3a is expressed in the same cell, it is also transported to the cell surface where it can bind S. The interaction between 3a and S enables both proteins to become internalized, resulting in a decrease in the expression of S on the cell surface. Thus, this viral-viral interaction confers the functional role for the YxxΦ motif found in other coronaviruses to the SARS-CoV S. This hypothesis also implies that the precise mechanisms used by TGEV and SARS-CoV to reduce the expression of S are different although in both cases, the YxxΦ motifs will be crucial. In TGEV, the YxxΦ motif in S caused it to be retained intracellularly, while in SARS-CoV, S that is transported to the cell surface becomes internalized again after it interacts with the 3a protein.

## Testing the hypothesis

Using mammalian cell culture system and biochemical methods, it will be possible to determine the exact effects of 3a on the trafficking properties of S. Mutagenesis studies can be used to map the protein domains that are important for the interaction between 3a and S and for the defining the manner by which 3a contributes to the reduction of cell surface expression of S. Given that a full-length infectious clone of SARS-CoV has been assembled [23], the use of reverse genetics would certainly reveal more about the interplay between 3a and S during SARS-CoV infection.

## Implication of the hypothesis

This hypothesis, if proven, will indicate that the interaction between SARS-CoV-unique 3a protein and S results in a reduction of S on the cell surface through the endocytotic properties of 3a [13]. During SARS-CoV infection, expression of S on the cell surface of an infected cell mediates fusion with un-infected neighboring cells, leading to syncytium formation. It follows that reducing the cell surface expression of S will delay this cell-damaging effect and prevent the premature release of unassembled viral RNA. It may also enhance virus packaging as it appears that the assembly of coronavirus occurs intracellularly, probably in the intermediate compartments between the endoplasmic reticulum and Golgi apparatus [24]. Clearly, this has certain advantages for the virus at certain stages of its life cycle. In addition, a reduction in the cell surface expression of S may also help the infected cell evade the host defense system and reduce the production of anti-S neutralizing antibodies. Conversely, host or viral factors that disrupt the interaction between S and 3a would favor the expression of S on the cell surface and enhance cell-cell fusion, a process that is important for viral spreading.

Table 1 shows a comparison of the amino acid sequences of the cytoplasmic tails of the S protein of different coronaviruses, including SARS-CoV, which is distantly related to the established group 2 coronaviruses [25], as well as two recently identified novel human coronaviruses, HCoV-NL63 [26] and HCoV-HKU1 [27]. The YxxΦ motifs are clearly present in all group 1 coronaviruses and also in IBV, which belongs to group 3. However, no YxxΦ motif is present in SARS-CoV and MHV, both group 2 coronaviruses. In addition, there is a YGGR motif in the S protein of RtCoV and YxxH motifs in the S proteins of the other group 2 coronaviruses, BCoV, HEV and HCoV-HKU1. However, these motifs may not be able to function as signaling motifs because both R and H are not hydrophobic amino-acids. Therefore, HCoV-OC43 is the only one of these group 2 coronaviruses that encodes a S protein with a YxxΦ motif. It is still unclear how the localization of S is modulated in those viruses that lack YxxΦ motifs in the S proteins and further studies will be needed to understand the different signaling pathways that are important for regulating the trafficking properties of S. Indeed, the dilysine endoplasmic reticulum retrieval signal, which is a different type of sorting signal from the YxxΦ motif, in the cytoplasmic tail of IBV was reported to be important for intracellular retention of S [28].

**Table 1:** Amino acid sequences of the cytoplasmic tail of spike (S) proteins of coronaviruses are compared with the YxxΦ (where x is any amino acid and Φ is an amino acid with a bulky hydrophobic side chain) motifs found in SARS-CoV 3a protein and other cellular proteins that are known to undergo endocytosis.

Protein	Amino acid sequences in the cytoplasmic tail^a^
TGEV S^b^	TM-CLGSCCHSICSRRQFENYEPIEKVHVH
PRCoV S^b^	TM-CLGSCCHSIFSRRQFENYEPIEKVHVH
CCoV S^b^	TM-CLGSCCHSICSRGQFESYEPIEKVHVH
FCoV S^b^	TM-CLGSCCHSICSRRQFENYEPIEKVHVH
PEDV S^b^	TM-CCGACFSGCCRGPRLQPYEAFEKVHVQ
HCoV-229E S^b^	TM-CFASSIRGCCESTKLPYYDVEKIHIQ
HCoV-NL63 S^b^	TM-CLTSSMRGCCDCGSTKLPYYEFEKVHVQ
BCoV S^c^	TM-ICGGCCDDYTGHQELVIKTSHDD
HCoV-OC43 S^c^	TM-KCGGCCDDYTGYQELVIKTSHDD
HEV S^c^	TM-KCGGCCDDYTGHQEFVIKTSHDD
MHV S^c^	TM-KKCGNCCDECGGHQDSIVIHNISSHED
RtCoV S^c^	TM-KCGNCCDEYGGRQAGIVIHNISSHED
HCoV-HKU1 S^c^	TM-KCHNCCDEYGGHHDFVIKTSHDD
SARS-CoV S^c^	TM-GACSCGSCCKFDEDDSEPVLKGVKLHYT
IBV S^d^	TM-KKSSYYTTFDNDVVTEQYRPKKSV
SARS-CoV 3a^e^	TM-38aa-YNSVTDTIVVTEGD-101aa
TfR^e^	19aa-YTRFSLARQVDGDNSHV-26aa-TM
LDLR (proximal)^e^	TM-17aa-YQKTTEDEVHICH-20aa
LDLR (distal)^e^	TM-34aa-YSYPSRQMVSLEDDVA
CD-M6PR^e^	TM-34aa-YRGVGDDGLGEESEERDDHLLPM
ASGPR^e^	MTKEYQDLQHLDNEES-24aa

^a^Sequences were obtained from National Center for Biotechnology Information (NCBI). Yxxx tetrapeptides are underlined and abbreviations used are: TM, transmembrane domain, aa, amino acids.

^b^S proteins of group 1 coronaviruses: TGEV, transmissible gastroenteritis virus (AJ271965); PRCoV, porcine respiratory coronavirus (Z24675); CCoV, canine coronavirus (D13096); FCoV, feline coronavirus (AY204704); PEDV, porcine epidemic diarrhea virus (AF353511); HCoV-229E, human coronavirus 229E (AF304460); HCoV-NL63, human coronavirus NL63(AY518894).

^c^S proteins of group 2 coronaviruses: BCoV, bovine coronavirus (AF220295), HCoV-OC43, human coronavirus OC43 (AY585228), HEV, porcine hemagglutinating encephalomyelitis virus (AY078417), MHV, murine hepatitis virus (AF201929), RtCoV, rat coronavirus (AF207551), HCoV-HKU1, human coronavirus HKU1 (AY597011), SARS-CoV, SARS coronavirus (AY283798).

^d^S protein of group 3 coronavirus: IBV, infectious bronchitis virus (M95169).

^e^SARS-CoV 3a protein (AY283798) and other cellular proteins that are known to undergo endocytosis. Abbreviations: TfR, transferrin receptor (P02786), LDLR, low-density lipoprotein receptor (P01130); CD-M6PR, cation-dependent mannose 6-phosphate receptor (P24668); ASGPR, asialoglycoprotein receptor (P07306).

It therefore appears that the cell surface expression of S protein of SARS-CoV can be reduced like that for other coronaviruses, but the mechanism may be different. The trafficking of SARS-CoV S may be mediated through 2 separate viral proteins, expressed from separate subgenomic RNA, and regulated by numerous complex cellular processes including the efficiency of transcription and translation, post-translation modification and stability of the viral proteins, as well as their interactions with host factors. Indeed, it is crucial to determine how this unique pathway benefits replication of the SARS-CoV. It is also interesting to note that sequence comparison of isolates from different clusters of infection showed that both S and 3a showed a positive selection during virus evolution [29,30], implying that these proteins play important roles in the virus life cycle and/or disease development and is consistent with the proposal that 3a has evolved to modulate the trafficking properties of the spike protein.

## Competing interests

The author(s) declare that they have no competing interests.

## Author's contributions

Yee-Joo Tan is responsible for the entire manuscript.
